# Prevalence of Asymptomatic Hyperuricemia and Associated Factors in Patients with Type 2 Diabetes Mellitus: A Cross-Sectional Study in Southern Vietnam

**DOI:** 10.3390/diagnostics16152413

**Published:** 2026-07-31

**Authors:** Kim Son Tran, Minh Phuong Vo, Minh Quan Duc Nguyen

**Affiliations:** Department of Internal Medicine, Faculty of Medicine, Can Tho University of Medicine and Pharmacy Hospital, 179 Nguyen Van Cu Street, Can Tho City 94000, Vietnam; tkson@ctump.edu.vn (K.S.T.); vmphuong@ctump.edu.vn (M.P.V.)

**Keywords:** asymptomatic hyperuricemia, type 2 diabetes mellitus, associated factors

## Abstract

**Background/Objectives**: Asymptomatic hyperuricemia is a common yet frequently overlooked condition in the management of patients with type 2 diabetes mellitus (T2DM), despite its potential association with cardiovascular events. This study aimed to determine the prevalence and associated factors of asymptomatic hyperuricemia among patients with T2DM. **Methods**: A cross-sectional study was conducted on 235 patients with T2DM at a tertiary hospital. Demographic, clinical, laboratory, and medication data were collected. Asymptomatic hyperuricemia was defined as a serum uric acid level ≥ 420 µmol/L in men or ≥360 µmol/L in women, in the absence of clinical symptoms of gout. Hyperuricemia was classified by severity, and logistic regression analysis was used to identify associated factors. **Results**: The prevalence of asymptomatic hyperuricemia was 31.1%, including 25.9% with mild hyperuricemia (serum uric acid 420–540 µmol/L in men and 360–540 µmol/L in women) and 5.1% with severe hyperuricemia (serum uric acid > 540 µmol/L). Independent associated factors included body mass index (adjusted OR: 1.18; 95% CI: 1.01–1.36; *p* = 0.032), alcohol consumption (adjusted OR: 6.05; 95% CI: 1.79–20.41; *p* = 0.004), and lower estimated glomerular filtration rate (eGFR) (adjusted OR: 0.96; 95% CI: 0.93–0.99; *p* = 0.012). Subgroup analyses based on sex, renal function, and alcohol use demonstrated that these factors remained significant. The area under the receiver operating characteristic (ROC) curve for the predictive model was 0.869 (95% CI: 0.818–0.920). **Conclusions**: Asymptomatic hyperuricemia is prevalent in patients with T2DM (31.1%) and is associated with modifiable risk factors such as overweight, alcohol consumption, and chronic kidney disease. Our findings suggest that serum uric acid assessment may be useful as part of a comprehensive cardiovascular–renal–metabolic risk evaluation in patients with type 2 diabetes mellitus. However, prospective studies are needed to determine whether routine screening improves clinical outcomes.

## 1. Introduction

Type 2 diabetes mellitus (T2DM) is a common chronic disease worldwide, strongly associated with cardiovascular and renal complications, which are the leading causes of mortality. According to the International Diabetes Federation (IDF), in 2021, an estimated 537 million adults were living with diabetes globally, and this number is projected to rise to 643 million by 2030 and 783 million by 2045. Additionally, approximately 541 million people were estimated to have impaired glucose tolerance, a high-risk condition for developing T2DM. In the same year, over 6.7 million deaths related to diabetes were recorded among individuals aged 20 to 79, highlighting the global health burden posed by this condition [1]. In clinical practice, cardiovascular risk assessment and management in patients with diabetes often focus on traditional risk factors such as hypertension, dyslipidemia, obesity, smoking, and glycemic control [2,3,4]. However, certain metabolic risk factors, including uric acid, have received less attention and are often overlooked by clinicians.

Hyperuricemia has long been recognized as a central factor in gout. Nevertheless, accumulating evidence suggests that uric acid also plays a direct role in the pathogenesis of metabolic disorders, chronic inflammation, endothelial dysfunction, and atherosclerosis. Numerous studies have reported that elevated serum uric acid levels are associated with hypertension, chronic kidney disease (CKD), metabolic syndrome, and increased cardiovascular risk [5,6,7,8].

In patients with T2DM, the relationship between uric acid and cardiovascular risk becomes more complex due to its interactions with insulin resistance, dyslipidemia, and CKD [9,10]. Epidemiological studies have shown that elevated serum uric acid levels are independently associated with coronary artery disease, stroke, insulin resistance, and cardiovascular mortality, even after adjustment for traditional risk factors [11]. In addition to its cardiovascular implications, recent studies have also proposed serum uric acid as an early marker of kidney damage in T2DM. Previous experimental studies suggest that uric acid may contribute to glomerular fibrosis and inflammation via oxidative stress and local inflammatory activation, thereby promoting a decline in estimated glomerular filtration rate (eGFR) [12,13,14,15]. Hence, previous experimental and epidemiological studies have suggested that asymptomatic hyperuricemia may not only be a cardiovascular risk marker but may also play a pathogenic role in diabetic kidney disease [14,15]. Despite this, serum uric acid is often considered an ancillary test in daily practice, especially among patients without clinical signs of gout, leading to underrecognition of asymptomatic hyperuricemia.

Asymptomatic hyperuricemia is a prevalent condition but is rarely detected or actively monitored. For instance, in the United States, while only about 3.9% of adults are diagnosed with gout, approximately 20% exhibit elevated uric acid levels [16]. In patients with T2DM, the omission of uric acid evaluation may result in an incomplete risk assessment for cardiovascular and renal complications. Studies have confirmed that asymptomatic hyperuricemia in this population is independently associated with poor cardio-renal outcomes. Specifically, it contributes to atherosclerosis and endothelial dysfunction and is considered a predictor of coronary artery disease and kidney failure [14,17,18,19,20]. In the context of current recommendations that emphasize a comprehensive, multifactorial approach to cardiovascular risk management in T2DM, understanding the prevalence and determinants of asymptomatic hyperuricemia holds important clinical value.

In Vietnam, evidence regarding asymptomatic hyperuricemia among patients with type 2 diabetes mellitus remains limited, particularly in the Mekong Delta region, where epidemiological data are scarce. Previous studies have mainly focused on the prevalence of hyperuricemia or gout in the general population, while comprehensive evaluation of asymptomatic hyperuricemia together with metabolic, renal, lifestyle-related factors, and subgroup analyses in patients with T2DM has rarely been reported. Therefore, this study aimed not only to determine the prevalence of asymptomatic hyperuricemia but also to identify its independent associated factors and explore whether these associations differ according to sex, renal function, and alcohol consumption. These findings may provide locally and regionally relevant evidence to support cardio–renal–metabolic risk assessment in Vietnamese patients with T2DM.

## 2. Materials and Methods

### 2.1. Study Design and Participants

This study employed a descriptive cross-sectional design with analytical components, aiming to determine the prevalence of asymptomatic hyperuricemia and its associated factors among patients with T2DM. The study was conducted at the University of Medicine and Pharmacy Hospital, Can Tho, Vietnam, from 2024 to 2025. This period corresponded to the predefined data collection timeframe specified in the study protocol and approved by the institutional ethics committee.

Eligible participants were adult patients diagnosed with T2DM according to the 2024 criteria of the American Diabetes Association (ADA), who attended the hospital for examination and treatment during the study period.

All eligible patients with T2DM who attended the University of Medicine and Pharmacy Hospital, Can Tho, for medical examination and treatment during the study period were consecutively screened and enrolled if they fulfilled the predefined inclusion criteria and did not meet any exclusion criteria.

The minimum sample size was calculated using the single-population proportion formula:

$$n=\frac{Z_{1-\frac{\alpha }{2}}^{2}.P_{1}\left(1-P_{1}\right)}{d^{2}}$$

where *Z* = 1.96 for a 95% confidence level, *p* = 0.35 based on the prevalence of hyperuricemia reported in a previous Vietnamese study, and d = 0.065 as the margin of error. The minimum required sample size was 207 participants. During the study period, 235 eligible patients were enrolled, exceeding the calculated minimum sample size. During the study period, all eligible patients meeting the study criteria were consecutively enrolled until the required sample size was exceeded.

### 2.2. Inclusion and Exclusion Criteria

**Inclusion criteria:**

Patients aged ≥ 18 years.

Diagnosed with T2DM based on the 2024 American Diabetes Association (ADA) criteria [21].

Availability of complete clinical and laboratory data, including serum uric acid levels.

**Exclusion criteria:**

Patients with secondary hyperuricemia due to underlying conditions such as advanced kidney failure, leukemia, multiple myeloma, hemolytic anemia, recent chemotherapy or radiotherapy, or acute alcohol intoxication.

Patients with a known history of gout or currently receiving urate-lowering therapy.

Patients with acute conditions that may affect serum uric acid levels, including recent infections, trauma, or surgery.

Use of medications that may influence serum uric acid levels, including SGLT2 inhibitors, losartan, probenecid, sulfinpyrazone, salicylic acid, ascorbic acid, phenylbutazone, thiazide diuretics, anti-tuberculosis drugs, estrogens, and anticancer agents.

Pregnant women.

Medical records with incomplete data required for analysis.

### 2.3. Data Collection and Variable Definitions

Data for this study were collected from two sources: (1) electronic medical records at the hospital and (2) direct interviews conducted by trained nurses or research personnel. The collected information included demographic characteristics, medical history, lifestyle habits, current medications, as well as clinical and laboratory parameters.

#### 2.3.1. Definition of Asymptomatic Hyperuricemia

Asymptomatic hyperuricemia was defined as a serum uric acid (SUA) level ≥ 420 µmol/L in males or ≥360 µmol/L in females, in the absence of any clinical manifestations of gout, such as gouty arthritis or tophi formation [22].

#### 2.3.2. Classification of Serum Uric Acid Levels

Based on SUA concentrations, patients were categorized into three groups:

- Normal uric acid: SUA < 420 µmol/L in males and <360 µmol/L in females
- Mild hyperuricemia: SUA between 420–540 µmol/L in males or 360–540 µmol/L in females
- Severe hyperuricemia: SUA > 540 µmol/L in both sexes

#### 2.3.3. Variables Collected

*Demographics:* Age (years at the time of enrollment) and sex (male or female).

*Body Mass Index (BMI):* Calculated as weight (kg) divided by the square of height (m^2^), analyzed as a continuous variable.

*Lifestyle factors:*

- Smoking status: classified as current smoker, former smoker, or never smoked.
- Alcohol consumption: was defined as intake exceeding 3 standard drinks/day in men or 2 standard drinks/day in women. One standard drink was equivalent to 40 mL of spirits, 125 mL of wine, or one 330 mL can of beer [23].

*Duration of type 2 diabetes*: Calculated from the time of diagnosis to the time of data collection, and classified into two categories: <10 years and ≥10 years

*Current antidiabetic medications:* Documented use of metformin, sulfonylureas, dipeptidyl peptidase-4 inhibitors (DPP-4i), insulin, or combinations thereof.

*History of cardiovascular and renal comorbidities: *

- Hypertension (previously diagnosed and/or on treatment)
- Coronary artery disease (including history of myocardial infarction, stenting, or ischemic heart disease)

*Biochemical parameters:*

- Fasting plasma glucose (FPG)
- Glycated hemoglobin (HbA1c)
- Lipid profile: total cholesterol, low-density lipoprotein cholesterol (LDL-c), high-density lipoprotein cholesterol (HDL-c), and triglycerides
- Blood urea and serum creatinine

*Renal function:* Estimated glomerular filtration rate (eGFR) was calculated using the Chronic Kidney Disease Epidemiology Collaboration (CKD-EPI) equation. eGFR was used to assess the degree of kidney function impairment and its relationship with serum uric acid accumulation.

*Dyslipidemia* was defined as a previous diagnosis of dyslipidemia, current use of lipid-lowering therapy, or newly diagnosed dyslipidemia according to the National Cholesterol Education Program Adult Treatment Panel III (NCEP ATP III) criteria, defined as the presence of at least one of the following abnormalities: total cholesterol > 5.2 mmol/L, triglycerides > 1.7 mmol/L, LDL-cholesterol > 3.4 mmol/L, or HDL-cholesterol < 1.0 mmol/L [24].

All laboratory tests were performed at the hospital’s central laboratory according to internal quality control protocols.

### 2.4. Statistical Analysis

Data analysis was performed using SPSS software version 23. Quantitative variables were presented as mean ± standard deviation or median with interquartile range, depending on data distribution. Categorical variables were summarized as frequencies and percentages.

The prevalence of asymptomatic hyperuricemia and its severity levels were reported with 95% confidence intervals (95% CI). Differences in proportions between groups were assessed using the Chi-square test or Fisher’s exact test, as appropriate.

Univariate logistic regression analysis was used to screen for factors associated with asymptomatic hyperuricemia. Variables with statistical significance or clinical relevance were included in the multivariate logistic regression model to identify independent associations. Results were reported as odds ratios (ORs) with 95% confidence intervals. A *p*-value < 0.05 was considered statistically significant.

Continuous variables were assessed for normality. Normally distributed variables were presented as mean ± standard deviation and compared using the independent samples t-test, whereas non-normally distributed variables were expressed as median (interquartile range) and compared using the Mann–Whitney U test.

To evaluate the discriminative ability of the multivariate logistic regression model in predicting asymptomatic hyperuricemia, the Receiver Operating Characteristic (ROC) curve was utilized. The area under the curve (AUC) was calculated to reflect the model’s performance in distinguishing between patients with and without asymptomatic hyperuricemia. An AUC value closer to 1 indicates better classification accuracy of the model.

Additionally, subgroup analyses were performed based on sex, alcohol consumption status, and kidney function (eGFR < 60 vs. ≥60 mL/min/1.73 m^2^) to assess the robustness of the findings.

### 2.5. Ethical Considerations

The study was approved by the Ethics Committee of Can Tho University of Medicine and Pharmacy (Approval No.: 24.255.HV-ĐHYDCT). All patient data were anonymized and used solely for research purposes. Given the retrospective and non-interventional nature of the study, informed consent was waived in accordance with the Ethics Committee’s regulations.

### 2.6. Use of Artificial Intelligence

Artificial intelligence was used solely for language editing and manuscript formatting support. It was not employed to generate data, perform data analysis, or interpret study results.

## 3. Results

### 3.1. Baseline Characteristics of the Study Population

Among the 235 patients with T2DM included in the analysis (Table 1), 40.9% were male. The mean age of the study population was 60.9 ± 13.5 years, and 59.6% had diabetes duration of 10 years or longer. The mean BMI was 23.8 ± 2.6 kg/m^2^. Common comorbidities included hypertension (59.6%), dyslipidemia (67.2%), and chronic kidney disease (12.3%). Regarding behavioral risk factors, 14.9% of patients were current smokers, and 22.6% reported alcohol consumption.

In terms of laboratory characteristics, the median HbA1c level was 8.0% (interquartile range: 6.8–9.9), and the median fasting plasma glucose level was 7.8 mmol/L (6.4–11.3). The median serum uric acid concentration was 315 µmol/L (255–421). The majority of patients were receiving metformin (76.6%), while the use of DPP-4 inhibitors, sulfonylureas, and insulin was 50.2%, 26.8%, and 33.2%, respectively.

The group with hyperuricemia had a significantly higher proportion of males compared to the normouricemic group (53.4% vs. 35.2%; *p* = 0.008). Body weight and BMI were also significantly higher in the hyperuricemic group (*p* < 0.01). Additionally, the proportions of current smokers and alcohol consumers were markedly higher in the hyperuricemic group compared to those without hyperuricemia (both *p* < 0.001). With regard to comorbidities, chronic kidney disease (CKD) was more prevalent in the hyperuricemic group (31.5% vs. 3.7%; *p* < 0.001), while the prevalence of hypertension, dyslipidemia, and coronary artery disease did not differ significantly between the two groups.

Biochemical parameters showed that patients with hyperuricemia had significantly higher serum urea and creatinine levels, and lower estimated glomerular filtration rate (eGFR), compared with those without hyperuricemia (all *p* < 0.001). Moreover, HDL-cholesterol and LDL-cholesterol levels were significantly lower in the hyperuricemic group (*p* < 0.001 and *p* = 0.007, respectively). No significant differences were observed in total cholesterol or triglyceride levels. HbA1c and plasma glucose levels were also significantly lower in the hyperuricemic group (*p* = 0.003 and *p* = 0.014, respectively). The use of antidiabetic medications—including metformin, DPP-4 inhibitors, sulfonylureas, and insulin—did not differ significantly between the two groups (Table 1).

### 3.2. Prevalence of Asymptomatic Hyperuricemia

In the overall study population, 73 patients (31.1%) had asymptomatic hyperuricemia, including 25.9% with mild hyperuricemia and 5.1% with severe hyperuricemia (Table 2). The prevalence and severity of hyperuricemia differed significantly between men and women (*p* = 0.021). Overall, men exhibited a higher prevalence of hyperuricemia than women, with a greater proportion of severe hyperuricemia observed among male participants (Table 2).

Overall, men exhibited a higher prevalence of hyperuricemia than women. Notably, the majority of female patients with hyperuricemia were classified as mild cases, whereas the proportion of severe hyperuricemia was higher among males. This sex-related disparity helps explain the role of gender observed in the subsequent regression analyses (Table 2).

### 3.3. Logistic Regression Analyses for Factors Associated with Asymptomatic Hyperuricemia

In the univariate logistic regression analysis (Table 3), male sex (OR = 2.11; *p* = 0.009), alcohol consumption (OR = 3.41; *p* < 0.001), smoking (OR = 3.70; *p* = 0.001), and higher BMI (OR = 1.16 per kg/m^2^; *p* = 0.009) were significantly associated with asymptomatic hyperuricemia. Lower LDL-C and HDL-C levels, higher serum urea and creatinine concentrations, lower eGFR, and lower HbA1c levels were also significantly associated with hyperuricemia. In contrast, hypertension, coronary artery disease, triglycerides, total cholesterol, plasma glucose, and age were not significantly associated with the outcome.

Variables that were statistically significant in the univariate analysis or deemed clinically relevant were included in the multivariate logistic regression model. After adjusting for potential confounders, alcohol consumption (adjusted OR = 6.05; 95% CI: 1.79–20.41; *p* = 0.004), body mass index (adjusted OR = 1.18 per kg/m^2^; 95% CI: 1.01–1.36; *p* = 0.032), and lower estimated glomerular filtration rate (eGFR) (adjusted OR = 0.96; 95% CI: 0.93–0.99; *p* = 0.012) remained independently associated with asymptomatic hyperuricemia. Other variables—including age, male sex, smoking, LDL-c, HDL-c, urea, creatinine, and HbA1c—were no longer statistically significant in the multivariate model (Table 4).

**Figure 1:**
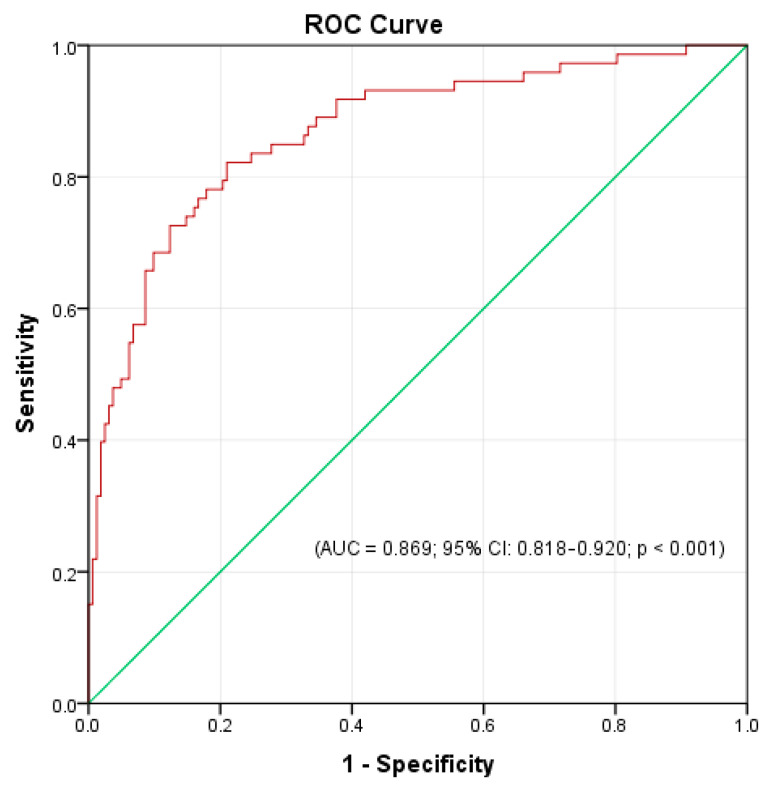
Receiver operating characteristic (ROC) curve of the final multivariable logistic regression model including sex, alcohol consumption, smoking status, BMI, LDL-c, HDL-c, urea, creatinine, eGFR, and HbA1c for predicting hyperuricemia (AUC = 0.869; 95% CI: 0.818–0.920; *p* < 0.001).

The receiver operating characteristic (ROC) curve was generated from the final multivariable logistic regression model presented in Figure 1, which included sex, alcohol consumption, smoking status, BMI, LDL-c, HDL-c, urea, creatinine, eGFR, and HbA1c as covariates. The model demonstrated good discriminative ability in distinguishing patients with and without asymptomatic hyperuricemia.

### 3.4. Subgroup Analyses

Subgroup analyses were performed to examine the robustness of factors associated with asymptomatic hyperuricemia. Multivariate logistic regression models were stratified by sex (male, female), estimated glomerular filtration rate (eGFR < 60 vs. ≥60 mL/min/1.73 m^2^), and alcohol consumption status.

In the subgroup analysis according to alcohol consumption (Table 5), among patients who did not consume alcohol, lower eGFR remained independently associated with asymptomatic hyperuricemia (adjusted OR = 0.95; 95% CI: 0.92–0.99; *p* = 0.016), while other variables were not statistically significant. In contrast, among alcohol users, LDL cholesterol was independently associated with asymptomatic hyperuricemia (adjusted OR = 0.50; 95% CI: 0.26–0.95; *p* = 0.035), whereas other variables—including sex, smoking, BMI, renal function, and HbA1c—did not show statistically significant associations.

In the subgroup analysis by sex (Table 6), among male patients, higher BMI (adjusted OR = 1.29; 95% CI: 1.01–1.64; *p* = 0.038) and smoking (adjusted OR = 6.26; 95% CI: 1.76–22.26; *p* = 0.005) were independently associated with asymptomatic hyperuricemia. Conversely, in female patients, lower eGFR remained independently associated with asymptomatic hyperuricemia (adjusted OR = 0.96; 95% CI: 0.92–0.99; *p* = 0.021), while other variables did not reach statistical significance.

In the subgroup analysis based on renal function (Table 7), among patients with eGFR ≥ 60 mL/min/1.73 m^2^, higher BMI (adjusted OR = 1.24; 95% CI: 1.06–1.44; *p* = 0.006) and higher serum creatinine levels (adjusted OR = 1.05; 95% CI: 1.02–1.07; *p* = 0.001) were independently associated with asymptomatic hyperuricemia. In contrast, no factors remained statistically significant in the subgroup of patients with eGFR < 60 mL/min/1.73 m^2^ after multivariable adjustment.

Subgroup analyses stratified by sex, alcohol consumption, and renal function are summarized in Table 5, Table 6 and Table 7. These exploratory analyses suggested potentially different patterns of association across clinically relevant subgroups. However, these findings should be interpreted with caution because several subgroup analyses were based on relatively small sample sizes, resulting in wide confidence intervals. Confirmation in larger prospective studies is warranted.

## 4. Discussion

Although previous international studies have reported the prevalence of hyperuricemia in patients with T2DM, evidence from Vietnam remains limited, especially regarding asymptomatic hyperuricemia and its associated clinical factors. The present study expands the existing evidence by providing data from a Vietnamese tertiary hospital, identifying independent associated factors after multivariable adjustment, and exploring these associations across clinically relevant subgroups.

Our study confirms that asymptomatic hyperuricemia is relatively common among patients with T2DM, with a prevalence of 31.1%. This aligns with global reports ranging from 10% to over 50%, depending on population and definitions. For example, Fennoun et al. reported 26.5%, and a large Chinese study found 21.24% (higher in males) [25,26], while African studies reported rates as high as 55.56% [27]. These differences likely reflect variations in demographics, lifestyle, and diagnostic thresholds. Our findings reinforce the global pattern of under-recognized hyperuricemia in diabetes. Consistent with prior studies, we observed a higher prevalence in males, attributed to estrogen’s uricosuric effects in premenopausal women [28]. Postmenopausal hormonal decline narrows this gap, explaining reports of similar or higher hyperuricemia rates in older diabetic women with metabolic syndrome [29,30].

BMI was a significant and independent predictor of hyperuricemia, supporting evidence that obesity-related insulin resistance impairs renal urate clearance and promotes reabsorption, leading to elevated uric acid [26,27]. Previous experimental studies have suggested that obesity-associated inflammation and increased expression of urate transporters (e.g., URAT1 and GLUT9) may contribute to elevated serum uric acid levels. However, these mechanisms were not evaluated in the present study [31,32]. Interestingly, dyslipidemia and LDL-c levels were higher in the non-hyperuricemic group. The reasons for this finding remain unclear. Differences in demographic characteristics, metabolic profiles, and lifestyle factors between the two groups may have contributed to this observation. These findings suggest that the relationship between lipid metabolism and serum uric acid levels may be complex and warrants further investigation. These observations suggest that the lipid–uric acid relationship is not strictly linear and may be shaped by treatment, metabolic status, and behavioral factors, requiring further investigation.

Our study revealed an inverse relationship between glycemic control and asymptomatic hyperuricemia. Higher HbA1c levels were associated with a lower risk of hyperuricemia in univariate analysis (OR ~0.84 per 1% increase, *p* < 0.01), though this association lost significance in multivariable analysis. Previous studies have proposed that glucosuria in poorly controlled diabetes may enhance urate excretion through renal mechanisms, whereas higher insulin levels may increase renal urate reabsorption. These biologically plausible mechanisms may explain the inverse association observed in our study; however, they were not directly evaluated in the present study and therefore should be interpreted cautiously. Our findings are consistent with Wei et al., who reported a reduced risk of hyperuricemia with increasing HbA1c (adjusted OR ~0.87) [33]. This highlights the need for cautious interpretation of uric acid levels in newly diagnosed or poorly controlled diabetic patients.

Conversely, reduced eGFR was a strong independent predictor of hyperuricemia (adjusted OR ~0.96 per 1 mL/min/1.73 m^2^ decrease, *p* < 0.05), consistent with previous evidence linking even mild renal impairment to decreased urate excretion [31]. Elevated serum creatinine also correlated with hyperuricemia in univariate analysis. This finding is consistent with previous studies reporting an association between reduced kidney function and elevated serum uric acid levels. Previous studies have suggested a bidirectional relationship between hyperuricemia and CKD; however, this mechanism was not directly evaluated in our cross-sectional study. Importantly, we excluded patients with advanced renal failure or secondary hyperuricemia, emphasizing that the observed association reflects the impact of early-stage CKD in T2DM [34].

Alcohol consumption emerged as an independent risk factor for asymptomatic hyperuricemia. Previous experimental and epidemiological studies suggest that alcohol consumption may increase uric acid production and reduce urate excretion through several biological mechanisms [35]. In our study, regular alcohol use was associated with a markedly higher risk of hyperuricemia (adjusted OR ~6.05 vs. non-drinkers, *p* < 0.01), consistent with previous epidemiological data and a recent meta-analysis highlighting strong links between ethanol—particularly from beer and spirits—and elevated uric acid levels [36]. Clinically, these findings suggest that alcohol consumption should be considered during the comprehensive clinical assessment of patients with T2DM. In contrast, smoking showed a significant association with hyperuricemia in univariate analysis (OR ~3.7, *p* = 0.001) but lost significance in multivariate analysis (*p* = 0.11), suggesting confounding by factors like sex, alcohol use, or BMI. Male participants—who had higher smoking rates—also naturally exhibit higher uric acid levels, possibly explaining the initial association. These findings align with Fennoun et al., who also found no independent link between smoking and hyperuricemia in diabetic patients [25]. While smoking may indirectly affect urate metabolism through oxidative stress, current evidence does not support it as a direct contributor to hyperuricemia. Nonetheless, smoking cessation remains vital due to its broader cardiovascular and renal risks.

In this study, neither hypertension nor coronary artery disease showed significant associations with asymptomatic hyperuricemia (*p* = 0.313 and *p* = 0.681, respectively), even after multivariate adjustment. This contrasts with prior evidence linking hyperuricemia to elevated cardiovascular risk and hypertension development via mechanisms such as renal microvascular injury, activation of the renin–angiotensin system, and nitric oxide reduction [37]. The lack of association in our cohort may stem from the exclusion of patients on urate-altering medications (e.g., thiazides, losartan) and the high prevalence of hypertension (60%) among T2DM patients, which may have diluted its independent effect—particularly in the presence of overlapping contributors like reduced eGFR. Similarly, while hyperuricemia has been implicated in coronary artery disease and heart failure in other studies, no significant association was observed here, potentially due to sample size limitations or effective risk factor control. Nonetheless, given its links to metabolic syndrome components, asymptomatic hyperuricemia remains clinically relevant in T2DM—even when blood pressure and coronary disease are well-managed [19,38].

Exploratory subgroup analyses suggested potentially different patterns of association according to sex, renal function, and alcohol use. However, because several subgroups included relatively few participants, these findings should be interpreted cautiously and require confirmation in larger studies. Among men, higher BMI and smoking were independent predictors of asymptomatic hyperuricemia (OR ≈ 1.29 per kg/m^2^ and OR ≈ 6.26, *p* < 0.05), while in women, only reduced eGFR remained significant (OR ≈ 0.96; *p* = 0.021). One possible explanation is that sex-related behavioral and biological differences may contribute to these observations: men were more exposed to modifiable risks, whereas renal impairment—more prevalent in older, postmenopausal women—was the dominant factor. The absence of smoking among female drinkers (0%) further supports this divergence. These findings emphasize sex-specific risk profiling: men may benefit most from weight and smoking interventions, while in women, renal monitoring is key.

In patients with preserved renal function (eGFR ≥ 60 mL/min/1.73 m^2^), BMI and serum creatinine were independently associated with hyperuricemia (*p* < 0.01), highlighting metabolic factors as key drivers. In contrast, no significant predictors emerged in the CKD subgroup (eGFR < 60), likely due to the overwhelming effect of renal impairment—present in over 80%—and limited sample size (~29 patients). This suggests that in CKD, lifestyle factors exert less influence on uric acid levels, while in those with intact kidney function, weight and metabolic control are critical.

Alcohol-based subgroup analysis showed that among non-drinkers, reduced eGFR was the only significant predictor (OR = 0.95; *p* = 0.016). However, in drinkers, eGFR lost significance, and instead, an inverse association between LDL-c and hyperuricemia emerged (OR ≈ 0.50; *p* = 0.035). One possible explanation proposed in previous studies is that alcohol-induced hepatic dysfunction or nutritional factors may influence both LDL cholesterol and serum uric acid levels. Despite a modest alcohol-user sample (~22.6%), this result illustrates how ethanol metabolism can reshape uric acid risk profiles. Clinicians should consider alcohol history when assessing hyperuricemia in T2DM and tailor interventions accordingly.

This study has several limitations that should be noted. First, because of the cross-sectional design, causal relationships cannot be established and the findings should be interpreted as associations rather than causal effects. Second, specific data on dietary habits, physical activity levels, and alcohol consumption quantities were not collected in detail, which may confound the analysis. Third, the subgroup analyses were based on relatively small sample sizes and were not adequately powered to confirm subgroup-specific associations. Therefore, these findings should be considered hypothesis-generating and require validation in larger prospective studies. Lastly, the study was conducted at a single healthcare center, which may limit the generalizability of the findings to other populations. However, the study also has several notable strengths. It is among the few studies in Vietnam to systematically investigate the prevalence and associated factors of asymptomatic hyperuricemia, including multivariate regression and subgroup analyses, in patients with type 2 diabetes. Our sample size was also relatively robust, larger than that of previous studies conducted in our region, which enhances the reliability of the findings. The exclusion of secondary causes of hyperuricemia (such as gout or diuretic use) enhanced the accuracy of evaluating the relationship between metabolic factors and serum uric acid levels. Furthermore, exploratory subgroup analyses provided additional insights into the roles of individual factors in different clinical contexts, offering practical value for patient care.

## 5. Conclusions

In this study, the prevalence of asymptomatic hyperuricemia among patients with type 2 diabetes was 31.1%, including 25.9% with mild elevation and 5.1% with marked elevation. Independent factors significantly associated with hyperuricemia included higher BMI, lower eGFR, and alcohol consumption. Exploratory subgroup analyses suggested that the observed associations may differ according to sex, renal function, and alcohol consumption, although these findings require confirmation in larger studies. Routine assessment of serum uric acid may facilitate a more comprehensive cardio–renal–metabolic risk evaluation in patients with type 2 diabetes mellitus. Given the cross-sectional design, the observed associations should not be interpreted as evidence of causality. Future prospective studies are warranted to confirm these findings and evaluate their clinical implications. This study provides additional evidence from a Vietnamese population and contributes to the currently limited literature regarding asymptomatic hyperuricemia in patients with T2DM in Southeast Asia.

## Figures and Tables

**Table 1:** Baseline Characteristics.

	Total (*n* = 235)	Hyperuricemia Group (*n* = 73)	Non-Hyperuricemia Group (*n* = 162)	*p*
Age, years	60.86 ± 13.50	62.92 ± 14.83	59.94 ± 12.79	0.118 *
Sex, male, *n* (%)	96 (40.9)	39 (53.4)	57 (35.2)	0.008 ***
Height, m	1.60 (1.54–1.68)	1.62 (1.57–1.70)	1.60 (1.52–1.66)	0.018 **
Weight, kg	61 (55–70)	65.0 (60.0–72.0)	60.0 (53.0–68.25)	0.001 **
BMI, kg/m^2^	23.80 ± 2.62	24.48 ± 2.42	23.50 ± 2.66	0.008 *
Duration of diabetes, ≥10-years, *n* (%)	140 (59.6)	45 (61.6)	95 (58.6)	0.664 ***
Smoker, *n* (%)	35 (14.9)	20 (27.4)	15 (9.3)	<0.001 ***
Alcohol drinker, *n* (%)	53 (22.6)	28 (38.4)	25 (15.4)	<0.001 ***
Hypertension, *n* (%)	140 (59.6)	47 (64.4)	93 (57.4)	0.313 ***
Dyslipidemia, *n* (%)	158 (67.2)	45 (61.6)	113 (69.8)	0.220 ***
CKD, *n* (%)	29 (12.3)	23 (31.5)	6 (3.7)	<0.001 ***
CAD, *n* (%)	54 (23)	18 (24.7)	36 (22.2)	0.681 ***
TC, mmol/L	5.21 (4.16–6.42)	4.85 (3.96–6.115)	5.415 (4.26–6.5225)	0.106 **
TG, mmol/L	2.44 (1.73–3.85)	2.65 (1.905–4.365)	2.33 (1.6775–3.7075)	0.253 **
LDL-c, mmol/L	2.99 (2.03–4.01)	2.60 (1.73–3.61)	3.175 (2.255–4.2275)	0.007 **
HDL-c, mmol/L	1.12 (0.97–1.35)	1.05 (0.90–1.22)	1.16 (1.0075–1.4025)	<0.001 **
Urea, mmol/L	5.40 (4.30–6.70)	6.40 (4.85–8.45)	4.95 (4.00–6.00)	<0.001 **
Creatinine, μmol/L	67.7 (55.1–83.4)	85.0 (70.95–123.5)	60.45 (52.925–74.475)	<0.001 **
eGFR (mL/min/1.73 m^2^)	93.0 (74.0–103.6)	70.96 (47.38–95.51)	96.51 (85.58–105.60)	<0.001 **
HbA1c, %	8.0 (6.8–9.9)	7.60 (6.60–8.95)	8.35 (7.20–10.00)	0.003 **
Glucose, mmol/L	7.84 (6.41–11.30)	7.17 (5.94–9.97)	8.25 (6.63–11.49)	0.014 **
Acid uric, µmol/L	315 (255–421)	471.0 (426.5–532.0)	274.0 (235.25–320.25)	<0.001 **
Antidiabetic medications				
Metformin, *n* (%)	180 (76.6)	58 (79.5)	122 (75.3)	0.488 ***
DPP-4i, *n* (%)	118 (50.2)	41 (56.2)	77 (47.5)	0.221 ***
SU, *n* (%)	63 (26.8)	17 (23.3)	46 (28.4)	0.413 ***
Insulin, *n* (%)	78 (33.2)	24 (32.9)	54 (33.3)	0.945 ***

* Independent samples *t* test, ** Mann–Whitney U test, *** χ^2^ test. Abbreviations: BMI, body mass index; CKD, chronic kidney disease; CAD, coronary artery disease; TC, total cholesterol; TG, triglycerides; LDL-c, low-density lipoprotein cholesterol; HDL-c, high-density lipoprotein cholesterol; HbA1c, glycated hemoglobin; DPP-4i, dipeptidyl peptidase-4 inhibitor; SU, sulfonylurea; eGFR, estimated glomerular filtration rate.

**Table 2:** Prevalence and severity of hyperuricemia stratified by sex (n = 235).

Uric Acid	Male (*n* = 96)	Female (*n* = 139)	Total (*n* = 235)
Normouricemia	57 (59.4%)	105 (75.5%)	163 (68.9%; 95% CI: 63.0–75.0)
Mild hyperuricemia	32 (33.3%)	29 (20.9%)	61 (25.9%; 95% CI: 19.9–31.2)
Severe hyperuricemia	7 (7.3%)	5 (3.6%)	12 (5.1%; 95% CI: 2.3–7.9)
*p*-value (Chi-square)	0.021		

**Table 3:** Univariate logistic regression analysis for factors associated with hyperuricemia.

Variable	Crude OR	95% CI	*p*
Age	1.02	0.996–1.038	0.118
Male sex	2.11	1.21–3.71	0.009
Hypertension	1.34	0.76–2.38	0.314
CAD	1.15	0.60–2.19	0.681
Alcohol use	3.41	1.81–6.44	<0.001
Smoking	3.70	1.77–7.75	0.001
BMI (per kg/m^2^)	1.16	1.04–1.30	0.009
Triglyceride (mmol/L)	1.04	0.95–1.13	0.395
Total cholesterol (mmol/L)	0.89	0.76–1.05	0.160
LDL-C (mmol/L)	0.77	0.62–0.95	0.015
HDL-C (mmol/L)	0.18	0.06–0.52	0.002
Urea (mmol/L)	1.41	1.22–1.62	<0.001
Creatinine (µmol/L)	1.04	1.03–1.06	<0.001
eGFR (mL/min/1.73 m^2^)	0.95	0.94–0.97	<0.001
HbA1c (%)	0.84	0.74–0.96	0.009
Glucose (mmol/L)	0.95	0.89–1.02	0.169

Abbreviations: CAD, coronary artery disease; BMI, body mass index; LDL-c, low-density lipoprotein cholesterol; HDL-c, high-density lipoprotein cholesterol; eGFR, estimated glomerular filtration rate; HbA1c, glycated hemoglobin.

**Table 4:** Multivariable logistic regression analysis for factors independently associated with hyperuricemia.

Variable	Adjusted OR	95% CI	*p*
Male sex	0.57	0.17–1.87	0.352
Alcohol use	6.05	1.79–20.41	0.004
Smoking	2.55	0.81–8.02	0.110
BMI (per kg/m^2^)	1.18	1.01–1.36	0.032
LDL-C (mmol/L)	0.85	0.64–1.14	0.287
HDL-C (mmol/L)	0.34	0.08–1.47	0.150
Urea (mmol/L)	1.20	0.96–1.49	0.102
Creatinine (µmol/L)	1.00	0.98–1.03	0.911
eGFR (mL/min/1.73 m^2^)	0.96	0.93–0.99	0.012
HbA1c (%)	0.90	0.77–1.06	0.220

Abbreviations: BMI, body mass index; LDL-c, low-density lipoprotein cholesterol; HDL-c, high-density lipoprotein cholesterol; eGFR, estimated glomerular filtration rate; HbA1c, glycated hemoglobin.

**Table 5:** Subgroup analysis stratified by alcohol consumption.

Variable	No Alcohol Adjusted OR (95% CI)	*p*	Alcohol Use Adjusted OR (95% CI)	*p*
Male sex	0.65 (0.18–2.39)	0.519	0.07 (0.001–7.10)	0.255
Smoking	3.34 (0.43–26.25)	0.252	1.94 (0.41–9.13)	0.400
BMI (kg/m^2^)	1.18 (0.98–1.41)	0.078	1.25 (0.92–1.70)	0.153
LDL-C (mmol/L)	1.02 (0.73–1.42)	0.927	0.50 (0.26–0.95)	0.035
HDL-C (mmol/L)	0.26 (0.05–1.39)	0.116	1.17 (0.04–38.25)	0.931
Urea (mmol/L)	1.27 (0.99–1.63)	0.065	0.95 (0.51–1.78)	0.876
Creatinine (µmol/L)	1.00 (0.97–1.03)	0.894	1.01 (0.92–1.12)	0.791
eGFR (mL/min/1.73 m^2^)	0.95 (0.92–0.99)	0.016	0.95 (0.86–1.06)	0.367
HbA1c (%)	0.90 (0.74–1.10)	0.289	0.89 (0.64–1.25)	0.504

**Table 6:** Sex-stratified subgroup analysis for factors associated with hyperuricemia.

Variable	Male Adjusted OR (95% CI)	*p*	Female Adjusted OR (95% CI)	*p*
BMI (kg/m^2^)	1.29 (1.01–1.64)	0.038	1.17 (0.95–1.43)	0.137
LDL-C (mmol/L)	0.65 (0.40–1.06)	0.086	1.04 (0.72–1.51)	0.832
HDL-C (mmol/L)	0.21 (0.01–3.61)	0.279	0.38 (0.07–2.03)	0.257
Urea (mmol/L)	1.30 (0.86–1.97)	0.211	1.21 (0.91–1.63)	0.195
Creatinine (µmol/L)	1.05 (0.96–1.15)	0.264	0.99 (0.97–1.02)	0.606
eGFR (mL/min/1.73 m^2^)	0.99 (0.92–1.07)	0.815	0.96 (0.92–0.99)	0.021
HbA1c (%)	0.97 (0.74–1.27)	0.834	0.90 (0.73–1.12)	0.356
Smoking	6.26 (1.76–22.26)	0.005	—	—
Alcohol use	—	—	43.17 (1.36–1373.10)	0.033

**Table 7:** Subgroup analysis stratified by kidney function (eGFR < 60 vs. ≥60 mL/min/1.73 m^2^).

Variable	eGFR < 60 Adjusted OR (95% CI)	*p*	eGFR ≥ 60 Adjusted OR (95% CI)	*p*
BMI (per kg/m^2^)	0.80 (0.48–1.33)	0.389	1.24 (1.06–1.44)	0.006
LDL-C (mmol/L)	1.26 (0.47–3.41)	0.648	0.82 (0.61–1.12)	0.210
HDL-C (mmol/L)	0.00 (0.00–1.14)	0.055	0.47 (0.11–2.02)	0.309
Urea (mmol/L)	1.61 (0.87–3.01)	0.133	1.16 (0.91–1.49)	0.230
Creatinine (µmol/L)	0.99 (0.96–1.02)	0.443	1.05 (1.02–1.07)	0.001
HbA1c (%)	0.61 (0.33–1.13)	0.118	0.93 (0.79–1.10)	0.394

## Data Availability

The data that support the findings of this study are available from the corresponding author, Q.Đ.M.N., upon reasonable request. No publicly archived datasets were used or generated.

## Abbreviations

The following abbreviations are used in this manuscript:

T2DM	Type 2 Diabetes Mellitus
SUA	Serum Uric Acid
eGFR	Estimated Glomerular Filtration Rate
LDL-C	Low-Density Lipoprotein Cholesterol
HDL-C	High-Density Lipoprotein Cholesterol
BMI	Body Mass Index
OR	Odds Ratio
CI	Confidence Interval
CKD	Chronic kidney disease
